# Mitral Valve Replacement Under Venoarterial Extracorporeal Membrane Oxygenation Support for Severe Streptococcus Mitis Endocarditis

**DOI:** 10.7759/cureus.7556

**Published:** 2020-04-06

**Authors:** Talha Ahmed, Marianne Wallis

**Affiliations:** 1 Internal Medicine, University of Maryland Medical Center, Baltimore, USA; 2 Critical Care Medicine, University of Maryland, Baltimore, USA

**Keywords:** streptococcus mitis, endocarditis, mitral valve, mitral valve replacement

## Abstract

Streptococcus mitis is an alpha-hemolytic species of Streptococcus and is the most prevalent organism of the oral flora. In patients with dental procedures or trauma and underlying damaged heart valves, it can cause infection of the valves (endocarditis). It has a propensity to affect the left-sided heart valves (mitral and aortic valves) in non-intravenous drug users (IVDU), whereas right-sided heart valves (tricuspid and pulmonic valves) in IVDU. We describe a case of a patient who presented with mitral valve endocarditis that was treated with antibiotics. He was lost to follow-up and then presented four years after his index presentation in cardiogenic shock from severe streptococcal mitis endocarditis causing severe mitral regurgitation. His course deteriorated to the point where the patient required a valve replacement surgery under hemodynamic support.

## Introduction

Infective endocarditis (IE) is infection of the heart valves. It can involve either native valves or prosthetic valves. It is a condition with high mortality rate with an in-hospital mortality ranging from 15% to 22% and five-year mortality of approximately 40% [1]. Its diagnosis is based on the Duke criteria, which relies on the combination of findings from history, physical examination supported by blood cultures and echocardiogram. There are clear-cut guidelines regarding the medical and surgical management of IE. Patients who are treated medically usually require treatment for four to six weeks from the time when the cultures are negative [2,3]. Our patient who was initially treated with antibiotics during his index presentation was lost to follow-up and then later presented in cardiogenic shock four years later. His repeat echocardiogram showed a large mobile mitral valve (MV) vegetation involving almost entire posterior leaflet of the MV. Despite medical management, the cardiogenic shock worsened requiring mechanical circulatory support for valve replacement surgery with eventual recovery and discharge from the hospital. 

## Case presentation

**Figure 1:**
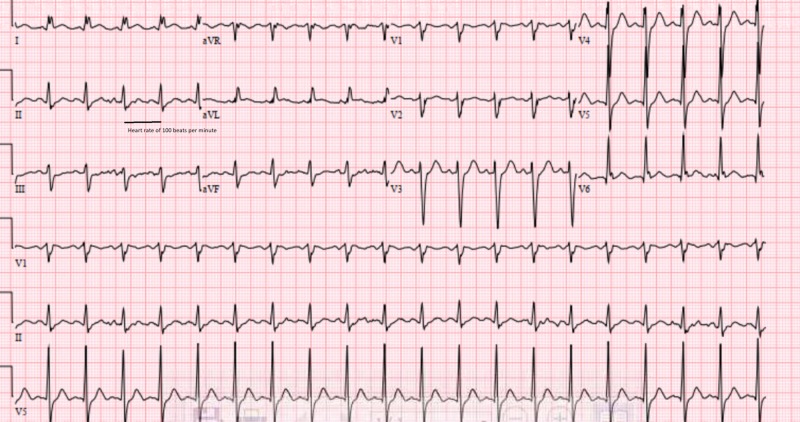
Electrocardiogram shows sinus tachycardia and incomplete left bundle branch block at presentation

A 39-year-old male with no reported history of drug use presented with chest pain, exertional shortness of breath and palpitations. His past history was significant for presentation to an outside facility for IE of MV complicated by acute septic emboli and intracranial hemorrhage four years back. At that time he was treated with appropriate antibiotics, demonstrated clearance of follow-up blood cultures and got a transesophageal echocardiogram revealing subcentimetic echodensity on posterior leaflet of MV with severe regurgitation and left ventricle ejection fraction (LVEF) of 55%. He was lost to follow-up and now presented after almost four years to our facility with current symptoms. Physical examination revealed crackles in bilateral lung fields and holosystolic murmur at the apical area radiating to the axilla. He progressively got hypotensive with systolic blood pressure of 50 mmHg requiring inotropic support with dobutamine and vasopressor support with norepinephrine. Electrocardiogram showed sinus tachycardia with incomplete left bundle branch block (Figure 1).

**Figure 2:**
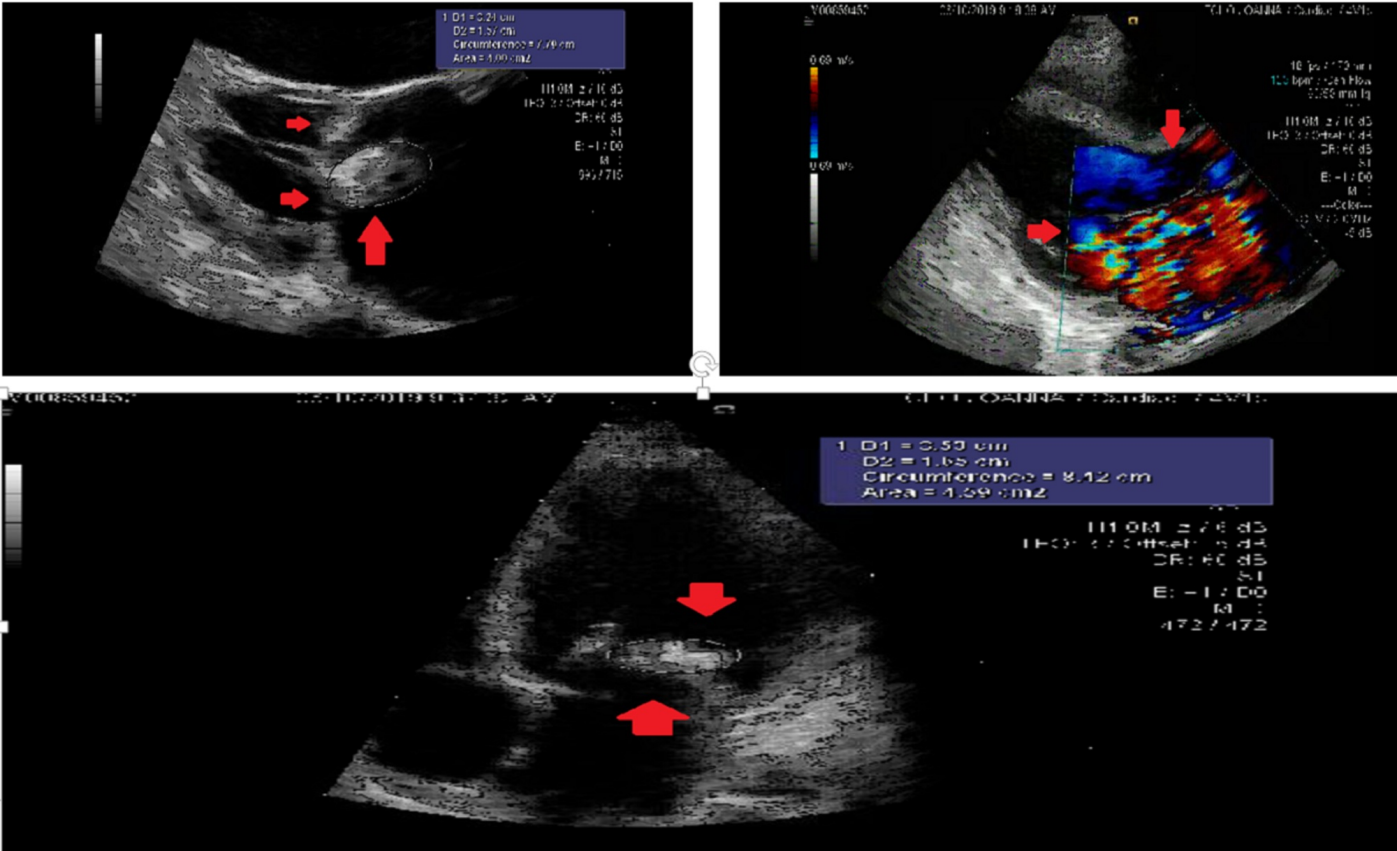
Transthoracic echocardiogram parasternal long axis view on the top showing mobile posterior leaflet vegetation (top left) and severe mitral regurgitation jet (top right), and at the bottom showing apical four chamber view with large vegetation on the posterior leaflet of mitral valve

A transthoracic echocardiogram (TTE) showed a large mobile vegetation along the entire posterior leaflet of MV with severe mitral regurgitation (MR) and an LVEF of 30% with dilated left atrium and ventricle. The right ventricular function was normal (Figure 2).

**Figure 3:**
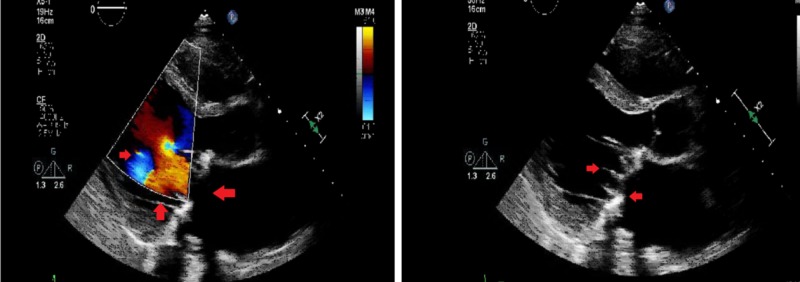
Transthoracic echocardiogram after valve replacement showing normal gradients across the replaced mitral valve that normally opens in diastole as well as dilated left atrium (left) and left ventricle (right)

He progressively developed signs of end-organ failure despite continuing antibiotics and continued to remain hypotensive despite being on maximum dose of vasopressors and inotropes. After careful consultation with cardiac surgery team, it was decided to cannulate him for venoarterial extracorporeal membrane oxygenation support (VA-ECMO) using femoral artery and femoral vein approach for inflow and outflow cannulas. The patient underwent valve repair surgery under VA-ECMO support. Follow-up TTE, the next day showed normal gradients across the replaced valve with LVEF of 15% with severe dilation and dysfunction (Figure 3).

The patient showed good recovery afterwards, and we were able to wean the vasopressors and inotropes. He was decannulated from ECMO and successfully discharged to a rehabilitation facility to complete his antibiotic course of six weeks for IE.

## Discussion

IE is a rare disease that carries a significant impact. In non-intravenous (IV) drug addicts, Streptococcus mitis causes left-sided endocarditis and in IV drug addicts, it causes right-sided endocarditis. It is part of normal oral flora and dental infections including abscesses, which can lead to spread in blood and endocarditis [4]. Our patient presented with recurrent Streptococcus mitis endocarditis four years after index presentation of his IE treated with antibiotics but with lost to follow-up for severe MR at index presentation. 

Management of IE involves antibiotics except under certain special circumstances like fungal etiology, refractory endocarditis, valvular abscess, hemodynamic instability and heart failure, embolic phenomena (strokes, renal infarcts, mesenteric ischemia and pulmonary emboli) and very large vegetations (more than 10 mm) [5]. At his index presentation, our patient had severe MR and was appropriately referred for evaluation for valve surgery; however, he was lost to follow-up [6].

At current presentation, he progressively developed cardiogenic shock that theoretically resulted from recurrent endocarditis with underlying severe MR. Treatment of cardiogenic shock resulting from IE is usually surgical [7]. However, for his underlying clinical instability he required hemodynamic support to perform the valve repair surgery. He was also treated with antibiotics for six weeks starting from day when valvular replacement was performed.

Streptococcal mitis is part of oral flora (belongs to streptococcal viridans group). However, no known source of recurrent endocarditis was found at second presentation in our patient. The cardiomyopathy had however progressed, and the vegetation now involved the whole posterior leaflet. One can hypothesize the etiology of the current event to be a slow progression of unresolved initial endocarditis (four years back) [8]. Repeat echocardiogram was not done to demonstrate resolution of subcentimetric vegetation that was found at his index presentation, and the antibiotic treatment was tailored to microbial culture data. Unfortunately, he was lost to follow-up and hence could not undergo further evaluation for valve replacement until he presented four years later in cardiogenic shock. 

In our case, recurrent streptococcal mitis endocarditis led to severe MR with gradual development of global left ventricle hypokinesis and eventual shock. Due to this mixed picture of sepsis and cardiogenic shock, the patient appropriately required valve replacement under hemodynamic support to treat cause of cardiomyopathy and achieve source control of sepsis.

## Conclusions

Streptococcus mitis endocarditis in non-IV drug users usually involves the left side of heart. It results from dental trauma, instrumentation and poor dental hygiene and infections/abscesses, and patients with valvular heart abnormalities are more susceptible. Recurrent endocarditis with large vegetations on the same valve may represent either a new event or progression of inadequately treated index event. The patient can develop a state of circulatory collapse (from mixed cardiogenic and septic shock) eventually requiring mechanical circulatory support. Valve replacement surgery under hemodyamic support is an option in these cases to attain source control for sepsis and treat the etiology of underlying cardiomyopathy. If untreated, this can prove to be fatal as untreated or partially treated IE has a high in-hospital and post-discharge mortality rate. 
